# Accelerating Development of Benziamidazole-Class Proton Pump Inhibitors: A Mechanism-Based PK/PD Model to Optimize Study Design with Ilaprazole as a Case Drug

**DOI:** 10.3390/pharmaceutics13030392

**Published:** 2021-03-15

**Authors:** Ranran Jia, Fan Zhang, Ni Wu, Wen Xu, Huitao Gao, Bo Liu, Hongyun Wang

**Affiliations:** 1 Clinical Pharmacology Research Center, Peking Union Medical College Hospital, State Key Laboratory of Complex Severe and Rare Diseases, NMPA Key Laboratory for Clinical Research and Evaluation of Drug, Beijing Key Laboratory of Clinical PK & PD Investigation for Innovative Drugs, Chinese Academy of Medical Sciences & Peking Union Medical College, Beijing 100032, China; pumc_ranranjia@student.pumc.edu.cn (R.J.); pumc_zhangfan@student.pumc.edu.cn (F.Z.); wuni@pumch.cn (N.W.); pumc_wxf@student.pumc.edu.cn (W.X.); zhangyanbao@pumch.cn (H.G.); 2 Consultant Committee, Hubei Yinghan Pharmaceutical Ltd., Wuhan 430074, China; liub@tcd.ie

**Keywords:** proton pump inhibitors, pharmacokinetic/pharmacodynamic model, ilaprazole

## Abstract

Proton pump inhibitors (PPIs) are the mainstay for treatment of acid-related diseases. This study developed a mechanism-based pharmacokinetic (PK) and pharmacodynamics (PD) model with ilaprazole as case drug, so as to support and accelerate the development of novel PPIs. The model was established and verified using the PK and PD data from 26 subjects receiving 5 to 30 mg of ilaprazole and 22 subjects receiving the loading dose of ilaprazole 20 mg followed by 10 mg once daily for 2 days. The nonlinear mixed-effects modeling approach was performed for the PK/PD model. A two-compartment model with linear elimination and covariates (body weight and gender) described the observed data well. The relationship between plasma concentrations of ilaprazole and gastric acid pH was well quantified with individual variability, in which the synthesis and degradation of H^+^/K^+^-ATPase, the food effect, the circular rhythms of gastric acid secretion, and the irreversible inhibition of H^+^/K^+^-ATPase by ilaprazole were integrated. This PK/PD model well predicted the PK and PD profile of ilaprazole in healthy subjects and patients with duodenal ulcers receiving wide range dose regimens. The mechanism-based PK/PD model provided a potential strategy to accelerate the development of novel PPIs by waiving the unnecessary clinical trials.

## 1. Introduction

As one of the world’s most frequently prescribed medications, proton pump inhibitors (PPIs) are regarded as the mainstay for treatment of gastric acid-related disorders including gastro-esophageal reflux disease (GERD), *Zollinger Ellison* syndrome and helicobacter pylori infection. However, despite their undoubted benefit, available PPIs fail to provide prolonged action and may require several doses to obtain maximum acid suppression and symptom relief. Moreover, most PPIs exhibit high inter-patient variability and it is reported that about 40% of GERD patients display an inadequate response to available PPI [1]. Thus, novel PPIs are urgently needed, which can at least ideally provide potent and prolonged pharmacological action with lower inter-individual variability.

PPIs inhibit gastric acid secretion by selectively and irreversibly blocking the H^+^/K^+^-ATPase [2,3]. Except for the same molecular target and action of mechanism, the available PPIs consist of a benzimidazole ring and a pyridine ring in terms of chemical structure. With regards to metabolism, PPIs are metabolized, at different extents, by CYP2C19 and CYP3A. Despite some inherent differences, available PPIs exhibit common features in PK and PD. Therefore, it is possible to set up mechanism-based PK/PD models for PPIs to capture the PK and PD properties. For a new PPI drug candidate, the PK/PD modeling and simulation can be used to optimize dose design and avoid the unnecessary clinical trials (e.g., dose group with poor efficacy or reaching the dose ceiling effect). 

Up to date, few PK/PD models are available for PPIs evaluation because the intragastric acid level is affected by complicated factors (e.g., the synthesis and degradation of H^+^/K^+^-ATPase, the circadian rhythm of gastric acid secretion, the dilution of food effect, nighttime break-through), apart from action of the drug. The first PK/PD model was proposed by Puchalski et al. for lansoprazole in which the drug effect, as well as the circadian rhythm and food effects on intragastric pH, were integrated [4]. Liu et al. extended the model to esomeprazole in Chinese [5]. However, the two models did not take into account the individual variability of PD index of PPIs; mechanistic-based PK/PD models consistent with the real case are required.

Ilaprazole is a novel PPI which also belongs to a class of substituted benzimidazole compound, chemically related to omeprazole [6,7]. Ilaprazole was predominantly metabolized by CYP3A and partly by CYP2C19 [8] and hence was not significantly influenced by CYP2C19 polymorphism [9,10]. Currently, ilaprazole has been approved for the treatment of gastric and duodenal ulcer, as well as erosive oesophagitis in China and South Korea as a highly effective and safe PPI in the treatment of acid-related disease [11,12]. The dosage regimen of intravenous ilaprazle is a loading dose of ilaprazole 20 mg followed by 10 mg once daily for peptic ulcer [11]. By our research team, the PK, PD and safety of intravenous ilaprazole have been investigated in healthy Chinese volunteers and patients with duodenal ulcers [11,13,14]. It was demonstrated that ilaprazole exhibited linear pharmacokinetics over the dose of 5 to 20 mg; the potency of intragastric acid pH control and onset of action were closely related to the dose and ilaprazole 20 mg provided the most significant effect in healthy subjects. This raises a question: is 20-mg the “right” dose producing the best effect? It is known that there is a “ceiling effect” of PPIs on the inhibition of H^+^/K^+^-ATPase that is the irreversible inhibition will be saturated when the given dose is high enough to block all the released H^+^/K^+^-ATPase. Can we explore the saturated “ceiling effect” effect at higher dose by PK/PD modeling and prediction? Another question is: What is the optimum dose regimen in terms of safety and efficacy? Which regimen is the most effective and can maintain higher efficacy?

In the present study we aimed to (1) build a mechanism-based PK/PD model for ilaprazole; (2) apply the model to predict the plasma concentrations and intra-gastric acid pH in healthy subjects and patients with duodenal ulcers at different doses, especially for the recommended dosing regimens (a loading dose of ilaprazole 20 mg followed by 10 mg once daily); (3) subsequently confirm the dose level reaching “ceiling effect”; and (4) optimize the dose regimens to provide stable and sustained pH control effect. Considering the clear mechanism and common PK features, this model could adequately predict PK and PD properties after being updated, and thus support and accelerate the development of other novel PPIs.

## 2. Materials and Methods

### 2.1. Date Source

The dataset for internal fitting was obtained from the study of 16 healthy Chinese volunteers [13]. The demographics were as follows (mean ± SD, range): age, 24.9 ± 4.1 (19–34) year; weight, 62.6 ± 8.0 (52.0–79.0) kg; height, 1.69 ± 0.08 (1.57–1.85) m; body mass index, 21.7 ± 1.3 (19.8–24.0) kg/m^2^. The volunteers were randomly divided into 4 groups (male: female, 1:1) for this randomized, open-label, cross-sectional trial. Each group was randomized to receive the following 4 dosing regimens in four sequences: a single oral dose of 10 mg with 200 mL of water or intravenous infusion of 5, 10 or 20 mg at 8:00 a.m. after overnight fasting. The washout period lasted 1 week before the next administration. Drinking water was not allowed until 2 h after the dose and standard meals were provided at 4 and 10 h on each study day. Nighttime was defined as a period between 20:00–8:00 the next day. Serial plasma samples were collected and intra-gastric pH was recorded within 24 h.

### 2.2. Methodology

**Figure 1:**
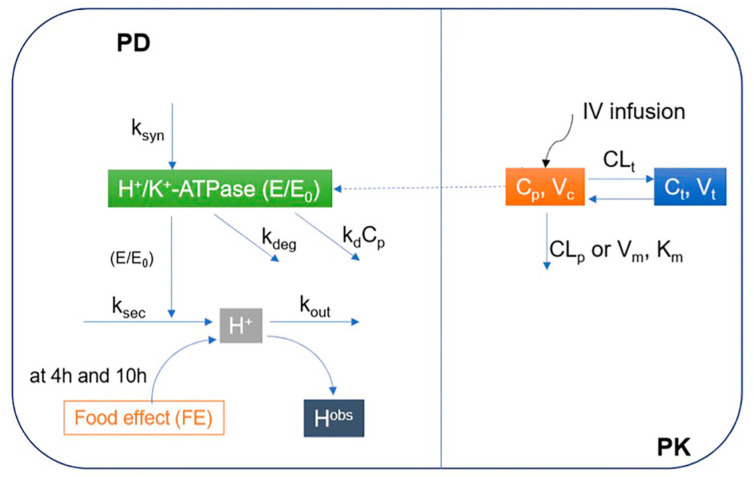
Proposed mechanism-based pharmacokinetic/pharmacodynamics (PK/PD) model of ilaprazole in Chinese volunteers. PD: Pharmacodynamics; PK: Pharmacokinetic; E/E0: Relative baseline H^+^/K^+^-ATPase activity; ksyn: H^+^/K^+^-ATPase synthesis rate constant; kdeg: H^+^/K^+^-ATPase degradation rate constant; ksec: Secretion rate constants for intra-gastric H^+^ concentration; kout: Elimination rate constant for intra-gastric H^+^ concentration; kd: Irreversible inhibition efficacy of H^+^/K^+^-ATPase by ilaprazole; Cp: Concentrations in central compartments; Ct: Concentrations in peripheral compartments; Hobs: Observed concentration of H^+^; Km: Michaelis-Menten constant; Vm: Maximum rate of metabolite production; Vc: Distribution volume of central compartment; Vt: Distribution volume of peripheral compartment; CLp: Clearance from the central compartment; CLt: Clearance from the central compartment to the peripheral compartment; IV infusion: Intravenous infusion.

A two-step approach was applied to develop the proposed mechanism-based PK/PD model of ilaprazole in Chinese individuals. To avoid the identification issue, i.e., to avoid fitting too many variables at the same time, the PK and PD of ilaprazole were modeled in sequence. Firstly, the PK relationship between the dose and the plasma concentration of ilaprazole was explored, and then used to describe the relationship between plasma concentration of ilaprazole and intra-gastric pH values (Figure 1).

### 2.3. Pharmacokinetic Modeling

The population PK modeling was performed using a nonlinear mixed-effects modeling approach: R (version 3.6.3, R Foundation for Statistical Computing, Vienna Austria). To determine the optimal structural model, one- or two-compartment models with linear or nonlinear (Michaelis–Menten) elimination were investigated. The final model was selected based on the Akaike Information Criterion (AIC), goodness-of-fit plots (GOF), and the Visual Predictive Check (VPC). The PK of ilaprazole was well characterized by a 2-compartment model with linear elimination (Equations (1) and (2)).

(1) $$V_{C}\frac{{\mathrm{dC}}_{P}}{\mathrm{dt}}=-{\mathrm{CL}}_{P}\times C_{P}-{\mathrm{CL}}_{t}\times \left(C_{P}-C_{t}\right)\times \;C_{p}(0)\;=\;0\;$$

(2) $$V_{t}\frac{{\mathrm{dC}}_{t}}{\mathrm{dt}}={\mathrm{CL}}_{t}\times \left(C_{P}-C_{t}\right)\times \;C_{t}(0)\;=\;0\;$$

where C_p_ and C_t_ were the concentrations in central and peripheral compartments of ilaprazole; ${\mathrm{CL}}_{P}$ and ${\mathrm{CL}}_{t}$ respented the elimination of the central compartment and peripheral compartment; V_c_ and V_t_ were the distribution volumes of ilaprazole of centra compartments and peripheral compartments, respectively.

The random effect (between-subject variability, BSV) of PK parameters was assumed to be normally distributed with a mean of 0 and a variance of ω^2^ described by an additive model (Equations (3)–(6)). Covariates (body weight and gender) were considered in this structure PK model according to the former study [15].

(3) $${\mathrm{CL}}_{P}=\theta_{{\mathrm{CL}}_{P}}\times \exp\left(\eta_{{\mathrm{CL}}_{P}}\right)\times {\left(\theta_{\mathrm{sex}}\right)}^{\mathrm{sex}}$$

(4) $${\mathrm{CL}}_{t}=\theta_{{\mathrm{CL}}_{t}}\times \exp(\eta_{{\mathrm{CL}}_{t}})$$

(5) $$V_{c}=\theta_{V_{c}}\times \exp\left(\eta_{V_{c}}\right)\times {\left(\frac{\mathrm{WT}}{70}\right)}^{\theta_{\mathrm{WT}}}\;$$

(6) $$V_{t}=\theta_{V_{t}}\times \exp\left(\eta_{V_{t}}\right)$$

where θ was the typical value of the population parameteres and η was the BSV of the parameter, which quantified the deviation between the typical parameter value and the individual parameter value. $\theta_{\mathrm{WT}}$ and $\theta_{\mathrm{sex}}$ described the extent of the covariate effect on ${\mathrm{CL}}_{P}$ and $V_{c}$, respectively.

The residual unexplained variability (RUV) of $C_{P_{\mathrm{obx}}}$ was described by the proportional error model (Equation (7)), which was assumed to be a standardized normal distribution in the range from 0 to 1.

(7) $$C_{P_{\mathrm{obx}}}=C_{P}\left(1+\varepsilon_{C_{P}}\right)$$

where $C_{P_{\mathrm{obx}}}$ was the observed plasma concentrations of ilaprazole; $\varepsilon_{C_{P}}$ quantified the residual error, representing measurement error.

### 2.4. Pharmacodynamics Modeling

The developed mechanism-based PK/PD model of ilaprazole was derived from population modeling of PK/PD of lansoprazole and esomeprazole [4,5], with a nonlinear mixed effect modeling approach using MATLAB (version R2018b) based on the fitted population PK model. The PD model of ilaprazole integrated the synthesis and degradation of H^+^/K^+^ ATPase, the asymmetric pH circadian rhythm mechanism, the food effect and the irreversible inhibition of ilaprazole on the H^+^/K^+^ ATPase (Figure 1) based on an indirect irreversible reaction (IDR) mechanism [4,5]. IDR model could describe the delayed inhibition effect of drug plasma concentration on the H^+^/K^+^-ATPase.

According to a modified surge based on circadian rhythm mechanism [16,17] and the asymmetric circadian function proposed by Liu et.al [5], we optimized an asymmetric pH circadian rhythm mechanism through the mean baseline pH data fitting (Equation (8)).

(8) $$f_{\mathrm{circadian}}=\frac{1+\frac{\mathrm{MA}}{{\left(\frac{{\mathrm{MT}}_{\max}}{\mathrm{MW}}\right)}^{4}+1}}{1+\frac{\mathrm{MA}}{{\left(\frac{t-{\mathrm{MT}}_{\max}}{\mathrm{MW}}\right)}^{4}+1}}$$

where MT_max_ was the peak time of the night intra-gastric H^+^ surge, MA and MW were the amplitude and width of the night intra-gastric H^+^ surge. Mean baseline pH data was used to fit the asymmetric pH circadian rhythm mechanism as all subjects are healthy volunteers, and the MT_max_, MA, and MW were assumed to be the same for the whole group.

Gastric acid secretion was stimulated due to increased vagal activity, gastric distention and chemical reaction of food and its components with stomach and gastrointestinal mucosa when taking food [18,19]. The food will also dilute or neutralize the pH in the stomach directly [20]. In this study, the stimulation of food on gastric acid secretion can be neglected as PPIs directly act on H^+^/K^+^-ATPase, and only the dilution of food on gastric H^+^ concentration was considered (Equations (9) and (10)). A standard meal taken at 4 h and 10 h was treated as a bolus effect on H^+^ concentration due to its dilution effect as there was no information about the duration of meals.

(9) $$F_{e}=\{\begin{array}{c} 1\\ 1+{\mathrm{FE}}_{4h}\times e^{\left(-k_{\mathrm{FE}}\right)\times \left(t-4\right)}\\ 1+{\mathrm{FE}}_{4h}\times e^{\left(-k_{\mathrm{FE}}\right)\times \left(t-4\right)}+{\mathrm{FE}}_{10h}\times e^{\left(-k_{\mathrm{FE}}\right)\times \left(t-10\right)} \end{array}\;\;\begin{array}{c} t<4\\ 4\leq t<10\\ 10\leq t \end{array}$$

(10) $$H^{\mathrm{obs}}=\frac{H}{F_{e}}\times F_{e}\left(0\right)=1;$$

where F_e_ was the ratio of H to H^obs^, which represented the effects of food on gastric acid secretion; H^obs^ was the H^+^ concentration after a meal, where H was the H^+^ concentration without considering food effects; FE_4H_ and FE_10H_ represented the food effect of lunch and dinner; k_FE_ was the food effect elimination rate constant, which reflected the bolus effect to dilute H^+^ concentration in stomach induced by eating, as well as the elimination rate constant of food effects. When being fasting, the food effect on observed H^+^ concentration was assumed as 1, which reflected there was no dilution of H^+^ concentration in the stomach. In addition, the washout rates of food effects on H^+^ concentration at 4h and 10h were assumed to be the same and subject independent, and the closed form was used for food effect.

On the basis of indirect response model (IDR) [21] and cell-killing model [4], we used the following model to describe the delayed inhibition effect of drug plasma concentration on the H^+^/K^+^-ATPase (Equation (11)).

(11) $$\frac{d\left(\frac{E}{E_{0}}\right)}{\mathrm{dt}}=k_{\mathrm{syn}}-k_{\deg}\times \left(\frac{E}{E_{0}}\right)-k_{d}\times \left(\frac{E}{E_{0}}\right)\times C_{P}\;k_{\mathrm{syn}}=k_{\deg\;}\;\mathrm{at}\;\mathrm{steady}\;\mathrm{state}\;\frac{E}{E_{0}}\left(0\right)=1;$$

where E/E_0_ was the relative baseline H^+^/K^+^-ATPase activity; $k_{\mathrm{syn}}$ and $k_{\deg\;}$ represented the production and degradation rate constant of E/E_0_; $k_{d}$ represented the inhibition efficacy of ilaprazole in inhibiting H^+^/K^+^-ATPase irreversibly.

The dynamics of intra-gastric H^+^ concentration was described by the IDR model integrating the circadian rhythm of H^+^ secretion and the activities of H^+^/K^+^-ATPase (Equations (12) and (13)).

(12) $$\frac{d\left(H^{+}\right)}{\mathrm{dt}}=k_{\sec}\times \left(\frac{E}{E_{0}}\right)-k_{\mathrm{out}}\times H^{+}\;\;\;\;\;\;H\left(0\right)=H_{\mathrm{BASE}\;}$$

(13) $$k_{\sec}=k_{\mathrm{out}}\times H_{\mathrm{BASE}\;}\times f_{\mathrm{circadian}}$$

where $k_{\sec}$ and $k_{\mathrm{out}}$ repersented the secretion and elimination rate constants for intra-gastric H^+^ concentration; H_BASE_, the baseline H^+^ concentration, referred to the H^+^ concentration corresponding to the minimum pH of mean baseline pH profile.

Fitted by nonlinear mixed effect model, different surge parameters (MT_max_, MA, and MW) and food effect parameters (FE_4h_, FE_10h_, and k_FE_) were assumed as the typical value of the population parameter, while the BSV was considered for the parameters of H^+^/K^+^-ATPase ($k_{\mathrm{out}}$, $k_{d}$ and $k_{\deg}$).

(14) $$\mathrm{MW}=\theta_{\mathrm{MW}}\;\;\;\;{\mathrm{FE}}_{4h}=\theta_{{\mathrm{FE}}_{4h}}\;\;\;\;k_{\mathrm{out}}=\eta_{k_{\mathrm{out}}}\times \exp\left(\theta_{k_{\mathrm{out}}}\right)\;\mathrm{MA}=\theta_{\mathrm{MA}}\;\;\;\;{\mathrm{FE}}_{10h}=\theta_{{\mathrm{FE}}_{10h}}\;\;\;\;k_{d}=\eta_{k_{d}}\times \exp\left(\theta_{k_{d}}\right)\;{\mathrm{MT}}_{\max}=\theta_{{\mathrm{MT}}_{\max}}\;\;\;\;k_{\mathrm{FE}}=\theta_{{\;k}_{\mathrm{FE}}}\;\;\;\;k_{\deg}=\eta_{k_{\deg}}\times \exp\left(\theta_{k_{\deg}}\right)$$

The additive error model (Equation (15)) was used to describe the RUV of ${\mathrm{pH}}^{\mathrm{obs}}$ with both being assumed to be normally distributed in the range from 0 to σ^2^.

(15) $${\;\mathrm{pH}}^{\mathrm{obs}}=\mathrm{pH}+\varepsilon_{\mathrm{pH}}\;$$

### 2.5. Model Evaluation and Validation

The predictive performance of the mechanism-based PK/PD model was evaluated by goodness-of-fit plots (GOF) and the visual predictive check (VPC, *n* = 500). The data sets were simulated from the final PK and PD model parameter estimation, and the median, 5th and 95th percentiles of the simulation data were compared with the observation data.

To evaluate the reliability of the mechanism-based PK/PD model, the data set from an independent study was used for external validation. The data set was available from 10 healthy subjects who received 30 mg infusion of ilaprazole at 8:00 on day after an overnight fasting [11]. Further details could be referenced in the original study. The evaluation of model performance was based on whether the observed data fitted well with the simulated PK/PD results.

Another two independent clinical studies from 12 healthy volunteers and 10 patients with duodenal ulcers who received the loading dose of ilaprazole 20 mg followed by 10 mg once daily for 2 days were used to further verify the reliability of the mechanism-based PK/PD model [11]. The studies were multiple-dose studies in which the subjects (12 healthy subjects and 10 patients with duodenal ulcers) received ilaprazole 20 mg at 8:00 on day 1 after an overnight fasting, and followed by 10 mg once daily on day 2 and day 3. The plasma concentrations and intragastric pH monitored for 72 h of healthy volunteers and patients with duodenal ulcers were used for the validation. The simulated PK and PD data of 2000 subject (male: female = 1:1, the body weight: median 70 kg and range from 40 to 80 kg) with food effect were compared with the individual intragastric pH data to evaluate the reliability of simulated PK and PD data under the loading dose of ilaprazole 20 mg, followed by 10 mg for 2 days.

### 2.6. Investigation of the “Ceiling Effect” of Ilaprazole

The irreversible inhibition of H^+^/K^+^-ATPase of ilaprazole was saturated when dose was increased to 30 mg according to our previous study [11]. The comparation of simulated 72-h concentrations curves and intra-gastric pH curves after dosing ilaprazole 10 mg, 20 mg, 30 mg and 40 mg for 3 days (2000 subjects male: female = 1:1, the body weight: median 70 kg and range from 40 to 80 kg) was used to investigate the “ceiling effect” of ilaprazole.

### 2.7. Optimization of Dose Regimens

To evaluate the optimum regimens for clinic based on the time reaching maximum gastric acid inhibition, the 72-h concentration curves of ilaprazole and intragastric pH curves under four different regimens were compared. The exposure of ilaprazole and the 72-h intragastric pH curves of 2000 subjects (male: female = 1:1, the body weight: median 70 kg and range from 40 to 80 kg) were simulated under four different dosing regimens using the validated mechanism-based PK/PD model. The simulated dosing regimens included (1) ilaprazole 10 mg once daily for 3 days; (2) ilaprazole 20 mg followed by 5 mg once daily for 2 days; (3) ilaprazole 20 mg followed by 10 mg once daily for 2 days; (4) ilaprazole 20 mg once daily for 3 days. The optimal scheme was mainly based on the inhibition of intra-gastric pH and safety of ilaprazole.

## 3. Results

### 3.1. PK Model Development and Estimation

A total of 16 healthy volunteers (half of men and women) and 645 observations were included in the pharmacokinetic and pharmacodynamics modeling process. The data fitted well using a 2-compartment model with linear elimination and covariates (body weight and gender), which was found to describe kinetics better than the 1-compartment mechanism (AIC decreased by 375.186), nonlinear clearance (AIC decreased by 172.131) or no covariates (AIC decreased by 21.348). The effects of BSV on parameter (V_c_, V_p_, CL_p_, and CL_t_) and RUV of the model were best described using an additive model. The final estimated PK parameters based on all subjects were showed in Table 1. The relative standard error (RSE%) of all parameters was less than 50%, indicating that the model was fitted robustly. The fixed effect of weight on V_p_ was1.35 and the fixed effect of sex on the CL_p_ was 1.30.

**Figure 2:**
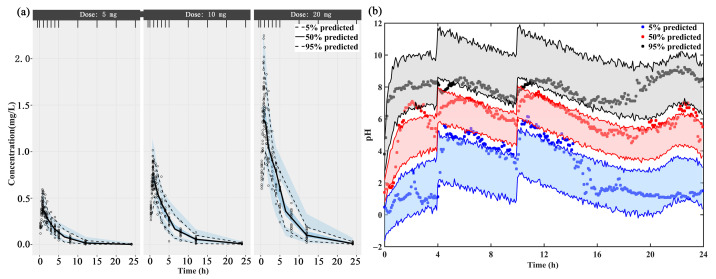
The visual predictive check (VPC) of (**a**) the final population PK model and (**b**) the mechanism-based PK/PD model. The upper, middle, and lower lines represent the 95th percentile, 50th percentile, and 5th percentile, respectively.

The individual concentration-time profiles of ilaprazole infusion confirmed the quality of PK model performance: the PK predictions overlapped with the observed plasma level in 3 dosage groups (5 mg, 10 mg and 20 mg) (Supplementary Figure S1a–c). The PK data of different dose groups were simulated into 500 data sets with the same experimental design and the obtained parameter estimates. The 5th, 50th and 95th percentiles (prediction intervals) and 90% confidence intervals of the simulation data are calculated and plotted as shadow areas corresponding to the observed concentrations (Figure 2a). The model fully predicted the observed data, and the prediction interval included most of the observed concentrations of ilaprazole.

### 3.2. PD Model Development and Estimation

Combining the asymmetric circadian secretion rhythm of gastric acid, food effects and drug effects, the PD parameters of ilaprazole were estimated and summarized in Table 2. The variabilities of H^+^/K^+^ ATPase were considered into this population PD model of ilaprazole, including its synthesis and degradation (k_deg_), the elimination of H^+^ (k_out_), and the irreversible inhibition of H^+^/K^+^ ATPase by ilaprazole (k_d_). The RSE% of all parameters was within the acceptable range. RUV of the model was best described using an additive error model.

The fitted 24-h intra-gastric pH were shown in Supplementary Figure S1d–f. The individual prediction and population prediction profiles were found to match the observed profiles well. The rise of pH value in stomach after taking ilaprazole was delayed. This delay was demonstrated by the IDR model well. The VPC plot demonstrated that 90% of the prediction interval overlaps the observation data, meaning the final model describes the observation data well (Figure 2b).

### 3.3. Model Evaluation and Validation

**Figure 3:**
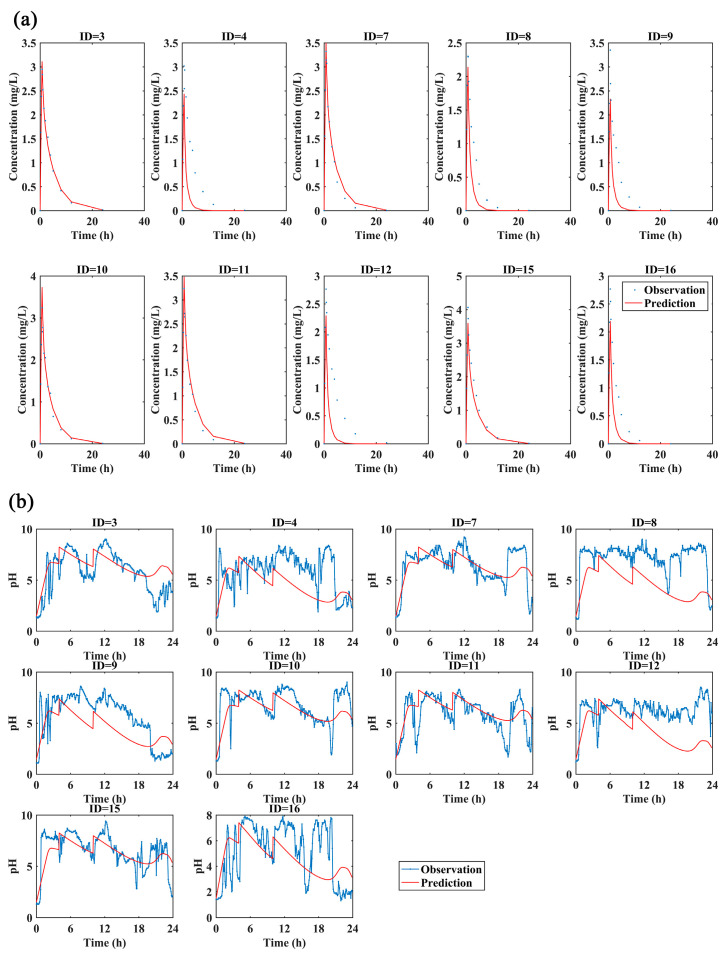
(**a**)The observed and predicted plasma concentration-time curves and (**b**) the observed and predicted pH profiles with 30 mg ilaprazole infusion. The predicted data for external validation of the PK and PD model overlaid with observations from 10 subjects individually (blue dots or lines: the observations; red lines: the predictions).

The PK and PD data of 10 healthy volunteers with 30 mg of ilaprazole infusion were used for the external validation. The observed data described well by the predicted PK/PD profiles (Figure 3a,b), indicating this model could predict the PK and PD data under different dosage.

**Figure 4:**
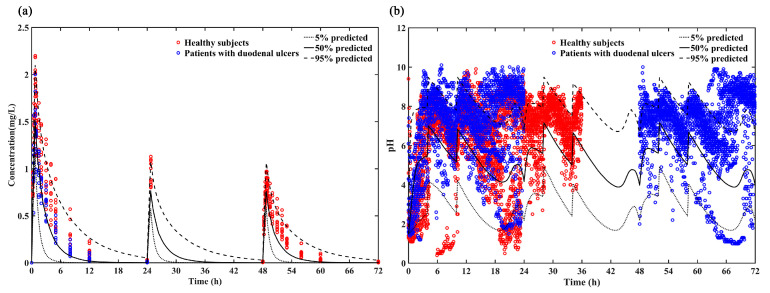
(**a**) Predicted PK profile (5%, 50% and 95% quantiles) vs. Observations of loading dose for healthy subjects (red circle) and patients with duodenal ulcers (blue circle); (**b**) Predicted intra-gastric pH profile (5%, 50% and 95%) vs. Observations of loading dose for healthy subjects (red circle) and patients with duodenal ulcers (blue circle).

The reliability of the model was evaluated by comparing the 5%, 50% and 95% quantiles of the predicted PK and PD results with the individual plasma concentrations (Figure 4a) and intragastric pH data (Figure 4b). Most of the individual plasma concentrations and intragastric pH data were within the 5th, 50th and 95th percentiles of the predicted pH values, indicating that this model provides a relatively accurate prediction in both healthy subjects and patients with duodenal ulcers for loading dose regimen (ilaprazole 20 mg followed by 10 mg for 2 days).

### 3.4. Investigation of the “Ceiling Effect” of Ilaprazole

**Figure 5:**
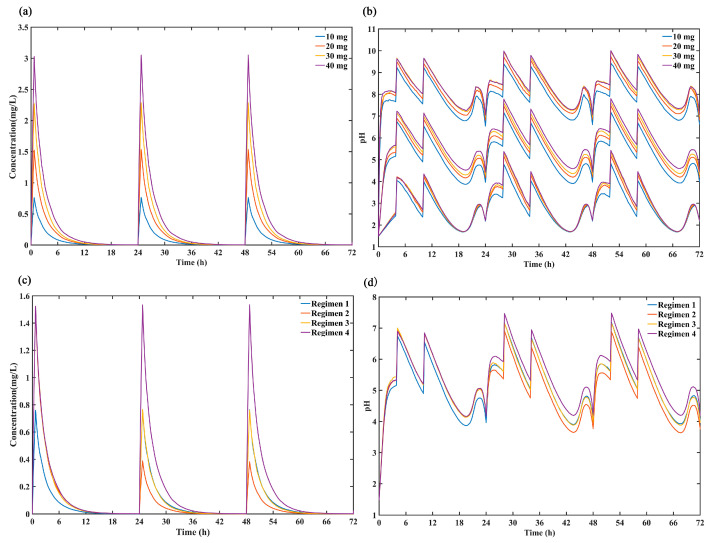
Comparison of the predicted (**a**) 50% percentiles of concentration profiles and (**b**) intragastric pH profiles (5%, 50% and 95% percentiles) after intravenous administration ilaprazole for 3 days of 4 dose group (10 mg, 20 mg, 30 mg and 40 mg). Comparison the simulated 50% percentiles of 24-h (**c**) concentration curves and (**d**) intra-gastric pH curves at 4 dose regimens (Regimen 1: ilaprazole 10 mg once daily for 3 days; Regimen 2: ilaprazole 20 mg followed by 5 mg once daily for 2 days; Regimen 3: ilaprazole 20 mg followed by 10 mg once daily for 2 days; Regimen 4: ilaprazole 20 mg once daily for 3 days).

In this study, the ceiling effect of ilaprazole was assessed by comparing the simulated 72-h concentration profiles (Figure 5a) and intragastric pH profiles (Figure 5b) after dosing ilaprazole 10 mg, 20 mg, 30 mg and 40 mg for 3 days. The results demonstrated that there was no significant difference in intragastric pH effect between 20 mg, 30 mg and 40 mg dosing while the plasma concentration increased proportionality over the dose range of 10–40 mg, which confirmed the dose ceiling effect of ilaprazole.

### 3.5. Optimization of Dose Regimens

The simulated plasma concentration time profiles for ilaprazole and intragastric pH curves under the different dosing regimens were shown in Figure 5c,d. It can be seen that there was no accumulation after 3 days of multiple infusions and the plasma concentration increased proportionality over the dose range of 5–20 mg. In addition, the regimen 4 (ilaprazole 20 mg once daily for 3 days) provided the strongest inhibition on gastric acid, and regimen 3 (ilaprazole 20 mg followed by 10 mg once daily for 2 days) was the second. There was no significant difference in gastric acid inhibition between regimen 1 (ilaprazole 10 mg once daily for 3 days) and regimen 3 (ilaprazole 20 mg followed by 10 mg once daily for 2 days) on days 2 and day 3 based on their same dosing regimen (10 mg once daily for day 2 and day 3).

## 4. Discussion

### 4.1. Establishment and Validation of the Mechanism-Based PK/PD Model

In this study, a mechanism-based PK/PD model of ilaprazole infusion was developed using the PK and PD data after a single dose of 5, 10 and 20 mg in healthy Chinese volunteers. A Pop PK model was used to characterize the PK of ilaprazole infusion for the first time. Based on the results of Pop PK analysis, we established a new mechanism-based PD model to describe the quantitative relationship between the exposure of ilaprazole and the intra-gastric pH value integrating the synthesis and degradation of H^+^/K^+^-ATPase, the irreversible inhibition of H^+^/K^+^-ATPase by PPIs, the effect of food, and the circadian rhythm of gastric acid secretion. Both internal and external validation showed that the model could accurately describe the inhibition of ilaprazole on gastric acid, as well as the food effect and circadian rhythm of gastric acid secretion.

Ilaprazole showed linear pharmacokinetics characteristics, which was consistent with the previous study. Wang et al. found that the peak plasma concentration and AUC of ilaprazole increased in proportion to the dose after taking 10, 20 or 40 mg ilaprazole daily for 5 days [22]. Another clinical study of 16 healthy Chinese subjects showed that the amount of plasma exposure to ilaprazole increased in proportion to the dose of 5–20 mg intravenously. Besides, the PK and PD of PPIs such as omeprazole, esomeprazole, lansoprazole, dexlansoprazole, and pantoprazole, are generally influenced by CYP2C19 genetic polymorphism [19,23,24]. However, previous studies had shown that the pharmacokinetics and pharmacodynamics of ilaprazole were not significantly influenced by the CYP2C19 polymorphism in healthy volunteers [10]. Another research indicated that ilaprazole metabolism was not relevant for CYP3A5 polymorphisms [9]. Incubation of ilaprazole with cDNA-expressed recombinant CYPs indicated that CYP3A was the major enzyme that catalyzes ilaprazole to ilaprazole sulfone [8]. However, it was reported that nonenzymatic sulfoxide reduction to ilaprazole sulfide was the major clearance pathway of ilaprazole in human, rather than CYP3A4-meditated sulfoxide oxidation [25]. Therefore, the polymorphism of CYP2C19 and CYP3A did not affect the PK of ilaprazole. Thus, this study did not consider the effect of gene polymorphism of CYP2C19 and CYP3A.

Second, this study quantified the contribution of the body weight and gender to plasma ilaprazole exposure and screened for the body weight as a significant covariate on $V_{c}\;\left(\theta \mathrm{wt}=1.35\right)$ as well as sex on ${\mathrm{CL}}_{p}\;\left(\theta \mathrm{wt}=1.30\right)$, which was consistent with the result of previous clinical research. The gender and body weight were selected as the significant covariates on ilaprazole ${\mathrm{CL}}_{p}$ and $V_{c}\;$(AIC decreased by 21.348). However, the covariates might have insignificant clinically effect on PK and PD of ilaprazole due to its safety and tolerance.

To explain the variability of H^+^/K^+^ ATPase, including its synthesis and degradation (k_deg_), the elimination of H^+^ (k_out_), and the irreversible inhibition of H^+^/K^+^ ATPase by ilaprazole (k_d_), a mechanism-based PK/PD model was constructed using NLME model. This PD model of ilaprazole could simulate the individual variabilities well and predicted the individual pH value in stomach accurately. The individual variability was considered into the mechanism-based population PK/PD model of PPIs for the first time. In addition, the food effect was treated as bolus effect on H^+^ concentration without duration of meals, which was explained for the difference in estimates (40.04 mL for EF_4h_ and 97.70 mL for EF_10h_) with the previous estimates (232–2312 mL). The estimated elimination of food effect (0.64/h) was comparable to those observed in the study by Puchalski et al. (0.79–0.98/h) and Liu et al. (0.694–1.05/h), respectively [4,5]. Besides, k_deg_ was estimated to be 0.15 1/h, which was discovered a profound discrepancy with the previous estimates in healthy Chinese adults (0.00109–0.00827 1/h) and in whites (0.04 1/h) [4,5]. This difference may be due to the significant individual differences in gastric pH values on baseline and after administration between individual. As a highly effective PPIs, k_d_ of ilaprazole was estimated as 18.24 (ng/mL)/h, which was higher than omeprazole (0.00388 (ng/mL)/h) and esomeprazole (0.0105–0.00505 (ng/mL)/h) [4,5,12]. The circadian rhythm parameters of gastric acid secretion could not obtain converged fitting when considering individual variation because of the complexity of circadian rhythm and the diversity of parameters.

### 4.2. Assessment of the Mechanism-Based PK/PD Model under a Wide Range of Dosing Regimens

Three independent studies were conducted to evaluate the predictive reliability of the model for healthy subjects and patients with duodenal ulcers after a wide range of dosing options. The mechanism-based PK/PD model was able to reasonably predict the PK and PD profiles after a higher single dose (30 mg) and the multiple doses of ilaprazole infusion (ilaprazole 20 mg followed by 10 mg for 2 days). The simulated stable and sustained pH control effect provided by the loading dose regimen was fitted well with the observed data, which indicating that the precise model is suitable for the recommended loading dose of ilaprazole 20 mg followed by 10 mg once daily for 2 days. Besides, due to the small difference in PK properties and similar intragastric acid inhibition effect between healthy subjects and patients with duodenal ulcers according to our previous study [11], the model can also predict the PK and PD characteristics after loading dose in patients with duodenal ulcers, which provide a new strategy for the development of PPIs.

### 4.3. Investigation of the Potential “Ceiling Effect” of PPIs

PPIs reduce intragastric acid secretion by inhibition of H^+^/K^+^-ATPase, and the inhibition will be saturated when the dose is high enough to block all the released H^+^/K^+^-ATPase. Before being saturated, the potency of intragastric acid pH control is closely related. Therefore, the dose reaching the “ceiling” saturation should be confirmed so that the best dosing regimen could be selected for patients.

In the present study, the established PK/PD model was successfully applied to evaluate the ceiling effect of ilaprazole. According to the simulation based on developed model, a dose ceiling effect was observed in agreement with hypothesis and trial data. The inhibition of ilaprazole on gastric acid reached the maximum effect at a single dose of 20 mg, which was completely consistent with the observation results of clinical trials. Wang et al. found that there was a saturated ceiling effect when dose was increased up to 30 mg, because the effect of ilaprazole 20 mg was comparable to that of 30 mg in both time-gastric pH curves and PD index (24-h pH values, the percentage of time with an intra-gastric pH > 6 et al.) [11,13]. To confirm the ceiling effect of ilaprazole completely, we simulated the gastric H^+^ concentration at dose of 10 mg, 20 mg, 30 mg and even higher dose of 40mg. It was found that there was no significant difference in the acid suppression for 20 mg, 30 mg, and 40 mg (Figure 5b). Based on the mechanism of action of benziamidazole-class PPIs, the saturated “ceiling” effect will be observed when given dose is high enough to block all the released H^+^-K^+^-ATPase. In the present manuscript, the PK/PD model accurately simulated the “ceiling effect” indicating the model could fit the mechanism of PPIs. For a new benziamidazole-class PPI drug candidate, application of the mechanism-based PK/PD mode can guide the dose design of subsequent clinical trials and avoid the unnecessary clinical trials.

### 4.4. Optimization of Dose Regimens

To achieve the stable and sustained pH control effect, 4 regimens about a loading dose of 10 or 20 mg on day 1 with different maintenance doses on subsequent 2 days were evaluated. On day 1 it was indicated that the regimen 1 (with a loading dose of 10 mg) was not enough to reach rapid onset of action. On the following 2 days, regimen 2 (with the maintenance dose of 5 mg) could not provide a sustained pH control effect as good as regiment 3 and 4. However, there were the high incidence of elevated activated partial thromboplastin time (APTT) and prothrombin time (PT) after a single dose of ilaprazole 10 mg and 20 mg while there was a slight increase in APTT after continuous infusion of ilaprazole 10 mg daily for 5 days [13,14]. Therefore, compared with regimen 3, the safety risk of regimen 4 (continuous administration of 20mg) was higher but with a slight difference in clinic effect. Overall, regimen 3 (a loading dose of ilaprazole 20 mg followed by 10 mg once daily for 2 days) may be the best regimen while considering both efficacy and safety.

Moreover, this mechanism-based PK/PD model could predict the PK and PD characteristics of ilaprazole under different situations, such as different dosages, with or without food effect, and population distribution (gender and body weight). Due to similar mechanism and common PK features, we suppose this model could also adequately predict PK and PD properties of other PPIs after being updated. It is possible to waive some clinical trials by PK/PD modeling and simulation, and thus accelerate the development of PPI drugs.

Several limitations should be recognized. For example, the food effect was considered to be the same for all subjects because of the same food intake in clinical trials for all volunteers. However, there was no time record for the subjects when drinking water. Clearly, according to the baseline pH profiles, some subjects never took food or drink outside the proposed meal time period.

## 5. Conclusions

To support the development of novel PPIs, a mechanism-based PK/PD model was established with ilaprazole as a case drug. This model quantified the relationship between the plasma concentration of ilaprazole and gastric pH, integrating the synthesis and degradation of H^+^/K^+^-ATPase, the irreversible inhibition of H^+^/K^+^-ATPase by PPIs and the effect of food, as well as the circadian rhythm of gastric acid secretion. This mechanism-based PK/PD model is suitable for both healthy subjects and patients with duodenal ulcers in a wide range of dosing options, especially for the loading doses of ilaprazole 20 mg followed by 10 mg for 2 days. A dose ceiling effect of ilaprazole was observed in agreement with hypothesis and trial data. The optimal dosing regimen for ilaprazole was evaluated according to the simulated efficacy and clinic safety.

For a new benziamidazole-class PPI drug candidate, based on the similar mechanism of action and common PK/PD features, this mechanism-based PK/PD model can provide a potential strategy to accelerate the development of novel PPIs by waiving the unnecessary clinical trials.

## Figures and Tables

**Table 1:** Summary of PK model parameters obtained by nonlinear mixed effect to all doses simultaneously.

Parameter	Unit	Definition	Estimate	95CI%	η
CL_p_	L/h	Clearance from the central compartment	2.57	(2.34, 2.81)	0.23
CL_t_	L/h	Clearance from the central compartment to the peripheral Compartment	4.59	(4.22, 5)	1.17
V_C_	L	Distribution volume of central compartment	8.80	(8.58, 9.02)	0.062
V_P_	L	Distribution volume of peripheral compartment	3.65	(3.63, 3.67)	0.29
θ_wt_		Fixed effect of body weight	1.35	(1.31, 1.39)	-
θ_sex_		Fixed effect of gender	1.30	(1.14, 1.48)	-
$\varepsilon_{C_{P}}$		Proportional residual error	0.12	-	-

95 CI%: 95% confidence interval. Values were converted back from mu-referencing.

**Table 2:** The mean ilaprazole pharmacodynamic parameters obtained by nonlinear mixed effects to all doses simultaneously.

Parameter	Unit	Definition	Estimate	RSE%	η
k_deg_	1/h	H^+^/K^+^-ATPase degradation rate constant	0.15	21.05	2.61
k_d_	(ng/mL)/h	Irreversible inhibition efficacy of H^+^/K^+^-ATPase by ilaprazole	18.24	12.24	1.38
		Elimination rate constant for intra-gastric H^+^ concentration			
k_out_	1/h	Baseline of intra-gastric H^+^ concentration	5.6	11.52	1.32
Base	mM	Width of the night intra-gastric H^+^ surge	0.033	Fixed	-
MW	h	Amplitude of the night intra-gastric H^+^ surge	0.99	30.18	-
MA		Peak time of the night intra-gastric H^+^ surge	28.48	3.28	-
MT_max_	h	Food effect of lunch	22.38	22.37	-
Ef_4h_	mL	Food effect of dinner	40.04	3.15	-
Ef_10h_	mL	Elimination rate constant for food effect	97.7	4.42	-
K_FE_	1/h		0.64	8.29	-
$\varepsilon_{C_{P}}$	-	Additive residual error	1.28	-	-

RSE, relative standard error.

## Data Availability

The data presented in this study are available within the article or Supplementary Material.

## Supplementary Materials

The following are available online at https://www.mdpi.com/1999-4923/13/3/392/s1, Figure S1: The ilaprazole plasma concentration versus time profiles for subjects after dosing 5 mg(a), 10 mg(b) and 20 mg(c) (blue lines, observed; red lines, individual fitted). And the individual 24-h intra-gastric pH profiles for subjects after dosing 5 mg(d), 10 mg(e) and 20 mg(f) (dots, observed; black lines, individual fitted; red lines, population fitted).
