# Supplementary material for: Accelerating Development of Benziamidazole-Class Proton Pump Inhibitors: A Mechanism-Based PK/PD Model to Optimize Study Design with Ilaprazole as a Case Drug
Source: Pharmaceutics. 2021 Mar 15;13(3):392. doi: 10.3390/pharmaceutics13030392 (PMC7998456; doi:10.3390/pharmaceutics13030392)
Supplement: Supplementary file 1 [file pharmaceutics-13-00392-s001.pdf]

pharmaceutics

MDPI

Supplementary Materials: Accelerating Development of Benziamidazole-Class Proton Pump Inhibitors: A Mechanism-Based PK/PD Model to Optimize Study Design with Ilaprazole as a Case Drug

Ranran Jia, Fan Zhang, Ni Wu, Wen Xu, Huitao Gao, Bo Liu and Hongyun Wang *

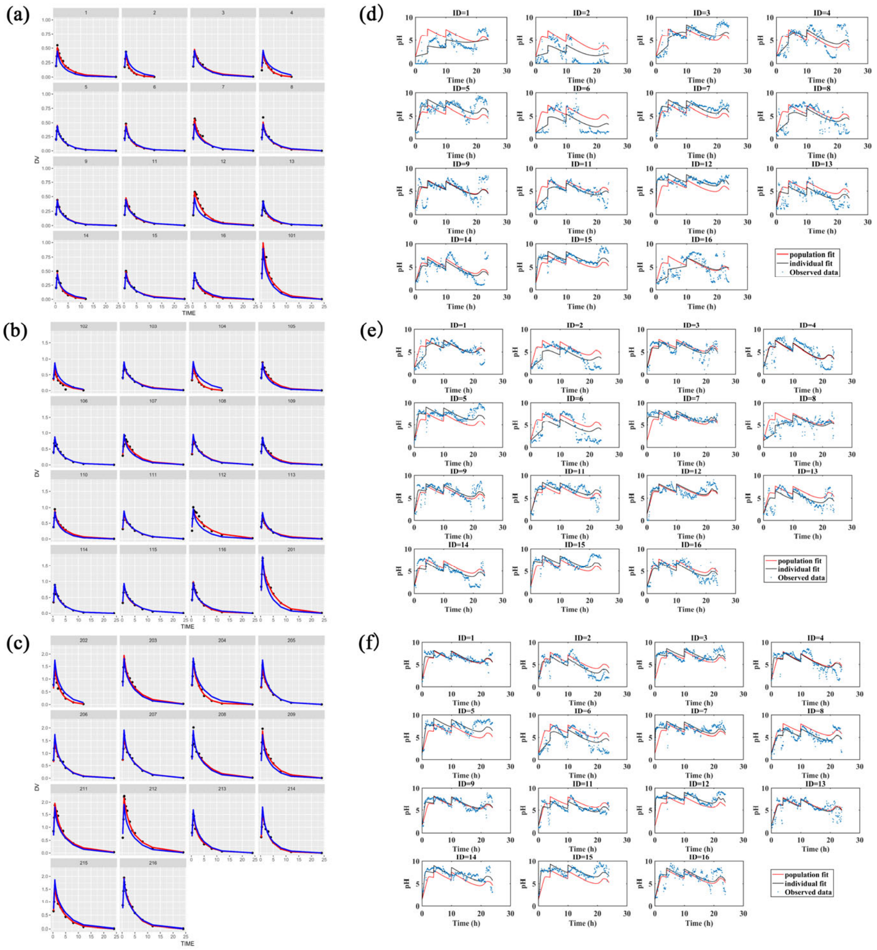

Figure S1. The ilaprazole plasma concentration versus time profiles for subjects after dosing 5 mg(a), 10 mg(b) and 20 mg(c) (blue lines, observed; red lines, individual fitted). And the individual 24-h intra-gastric pH profiles for subjects after dosing 5 mg(d), 10 mg(e) and 20 mg(f) (dots, observed; black lines, individual fitted; red lines, population fitted).
